# Systems modeling approaches for microbial community studies: from metagenomics to inference of the community structure

**DOI:** 10.3389/fmicb.2015.00213

**Published:** 2015-03-19

**Authors:** Mark Hanemaaijer, Wilfred F. M. Röling, Brett G. Olivier, Ruchir A. Khandelwal, Bas Teusink, Frank J. Bruggeman

**Affiliations:** ^1^Systems Bioinformatics, Amsterdam Institute for Molecules, Medicines and Systems, VU University AmsterdamAmsterdam, Netherlands; ^2^Molecular Cell Physiology, Amsterdam Institute for Molecules, Medicines and Systems, VU University AmsterdamAmsterdam, Netherlands

**Keywords:** microbial communities, metagenomic data integration, community modeling, genome-scale stoichiometric modeling, metabolism, flux balance analysis

## Abstract

Microbial communities play important roles in health, industrial applications and earth's ecosystems. With current molecular techniques we can characterize these systems in unprecedented detail. However, such methods provide little mechanistic insight into how the genetic properties and the dynamic couplings between individual microorganisms give rise to their dynamic activities. Neither do they give insight into what we call “the community state”, that is the fluxes and concentrations of nutrients within the community. This knowledge is a prerequisite for rational control and intervention in microbial communities. Therefore, the inference of the community structure from experimental data is a major current challenge. We will argue that this inference problem requires mathematical models that can integrate heterogeneous experimental data with existing knowledge. We propose that two types of models are needed. Firstly, mathematical models that integrate existing genomic, physiological, and physicochemical information with metagenomics data so as to maximize information content and predictive power. This can be achieved with the use of constraint-based genome-scale stoichiometric modeling of community metabolism which is ideally suited for this purpose. Next, we propose a simpler coarse-grained model, which is tailored to solve the inference problem from the experimental data. This model unambiguously relate to the more detailed genome-scale stoichiometric models which act as heterogeneous data integrators. The simpler inference models are, in our opinion, key to understanding microbial ecosystems, yet until now, have received remarkably little attention. This has led to the situation where the modeling of microbial communities, using only genome-scale models is currently more a computational, theoretical exercise than a method useful to the experimentalist.

## Introduction

Microbial communities are ubiquitous in nature and play key roles in the ecosystems of our planet. Humans depend on their activities as they play essential roles in element cycling and agriculture, e.g., through interactions between plants on the one hand and mycorrhiza and nitrogen fixing bacteria on the other hand (Fellbaum et al., 2012). Microbial communities are also exploited in food fermentations, e.g., in the production of cheese, yogurt, soy sauce, sauerkraut, and vinegar. Despite this major impact on human society, we still have little understanding of the design principles that determine microbial ecosystem functioning, robustness, evolution, and control. This means that the opportunities to rationally optimize the performance of such communities are currently very limited.

**Figure 1:**
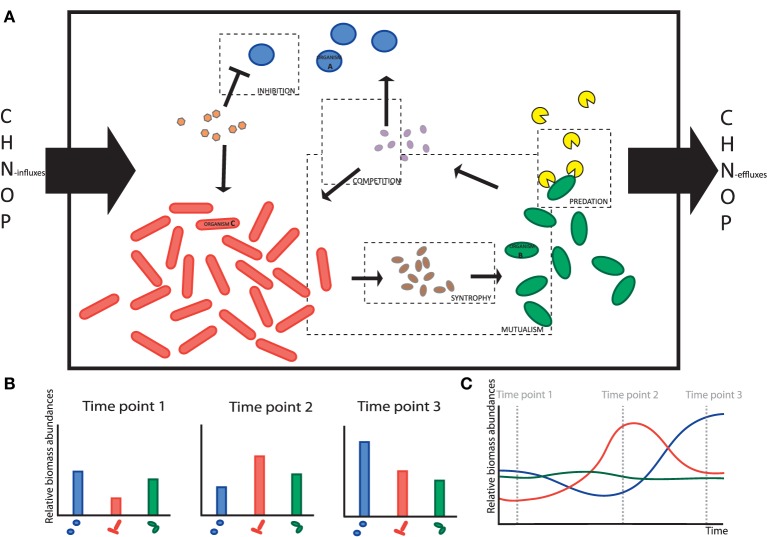
**Illustration of the diversity of a microbial ecosystem with various interactions. (A)** Is a schematic representation of an ecosystem with interactions among community members and the environment. The small particles are the metabolites, the big particles the community members, the boxes show the different interactions between community members. **(B)** Shows an example time series data set of the relative biomass abundances in this ecosystem. **(C)** Shows the real dynamics of the various species of the ecosystem in time. The mechanisms behind the dynamics cannot be captured by metagenomics time series data alone.

In view of the complexity of the systems we are dealing with, it should be clear that experimental data alone will not provide the desired understanding -regardless of how impressive current experimental techniques may be. For example, think about high-throughput DNA-, RNA and protein-sequencing that gives high resolution information on the identities of the occurring species, their (expressed) metabolic potentials and their (relative) abundances. Yet, the information gained from such meta-omics studies (we consider 16S rRNA gene sequencing to fall under meta-omics) is still relatively limited, as they only provide an indirect view into the metabolic activities of such microorganisms and virtually none about their relationship with the environment and each other. These properties must, in turn, be inferred from the data (see Figure 1, in which an ecosystem with various interactions between species is depicted). Typically, such data, at best, include metagenomics at different time points, revealing the differences in relative species abundances in the ecosystem. Methods such as stable isotope probing (SIP) (Dumont and Murrell, 2005), MAR-FISH (Lee et al., 1999) or nano-SIMS (Li et al., 2008) can provide additional and independent information on metabolites that are consumed by the various organisms in an ecosystem (He et al., 2012).

Inferring the metabolic activities of the species in a microbial community from the molecular data is largely an open problem in the field (Myrold et al., 2014). The huge challenge that microbial ecologists currently face is therefore how to infer the community state, i.e., the values of all the metabolite concentrations, species abundances and microbial activities (see also Glossary and below) from experimental data. A key question is therefore to determine what can and cannot be inferred from only metagenomic data and knowing this, what additional experimental measurements and computational methods are required to get a more complete understanding of a microbial ecosystem.

This challenge is to a large extent solved for single species where systems biology has delivered many methods for inference, data integration, and predictive modeling. These methods have become useful tools in more applied fields such as synthetic biology and metabolic engineering. Similarly, experimental methods such as MFA (see Glossary) (Toya and Shimizu, 2013) [e.g., isotopomer based (see Glossary) (Nöh et al., 2006)] and metabolomics (Wisselink et al., 2010) can be used to identify active metabolism of single species. Genome-based and genome-scale metabolic reconstructions can be used to integrate such data and help to understand and predict phenotypes of a microbial species (Bordbar et al., 2014; Long and Antoniewicz, 2014).

The question is to what extent these existing quantitative and computational approaches can be applied to microbial communities; several reviews and perspectives (Röling et al., 2010; Zengler and Palsson, 2012; Röling and Van Bodegom, 2014) pointed out the need for a more quantitative and systems based approach that can be used to understand microbial communities. In this contribution we will focus on the subsequent step: how to deal with quantitative data on ecosystem level? Our approach will be to investigate whether methods developed for monocultures, such as inference and quantitative modeling, are at all applicable for use in microbial ecosystems.

The single species methods we will describe have all been developed for the analysis of, and are therefore focused on, metabolism. One must therefore ask whether the microbial community state is dominantly shaped by metabolism-driven factors or whether it is also significantly dependent on factors unrelated to metabolism? One could distinguish two major classes of interactions between community members and the environment: those dictated by social traits and those driven by metabolism. We postulate that in many relevant cases, the metabolic component of the community is dominant. For instance, in glucose-fed biogas reactors the dominant species are all involved in the process of the metabolic conversion of glucose to methane (Fernandez et al., 2000). Many species-species interactions are metabolically driven, such as cross-feeding, nutrient competition, and predator-prey relations. All such metabolic processes account for mass flow through the ecosystem and the concomitant growth and turnover of microorganisms and metabolite levels.

However, communities are not solely structured by metabolic interactions. The non-metabolic interactions we exclude are social traits such as chemical warfare, bacteriocin production, quorum sensing, and other cell-to-cell interactions (either direct attachment, or other signaling mechanisms). We cannot exclude that social traits may play an important role in specific cases. Phages, for instance, can shape and alter whole ecosystems (Fuhrman, 1999), yet this important type of interaction cannot be covered by metabolism-based models. On the other hand, interactions such as quorum sensing also play a role in mono-cultures of *Escherichia coli* and *Pseudomonas aeruginosa*, however, model simulations suggests that quorum sensing has a minor effect on the phenotype of those single species (Oberhardt et al., 2008; Orth et al., 2011).

We will argue that limited quantitative experimental data, when combined with coarse-grained metabolic models, will be able to help researchers infer the community structure, i.e., the topology of species interactions (see Glossary for our definition of these often loosely defined terms). What do we mean by coarse-grained models? Basically, reduced complexity of the model. There are several ways to coarse-grain models, but in general it involves either ignoring parts of the system that are deemed less relevant, or lumping of many details of a subsystem into a higher-order description of that subsystem. A whole metabolic pathway can be lumped into one enzymatic reaction; the growth of a cell can be described by one Monod-type equation; a group of organisms can be summarized as one ecotype with an archetypical metabolic profile (“acetate consumer”). Such coarse-grained models can be gradually increased in detail over time. Parallel to these coarse-grained models, genome-scale metabolic models can be used as data repositories where new data and constraints are imposed on the models. These two approaches should in the end converge into the same description of the community. We will provide an example to illustrate how the combination of these approaches could aid in improving mechanistic understanding of microbial communities.

## Metabolic fluxes in single microorganisms: a largely solved inference problem

In metabolic networks, metabolites are produced and consumed by enzymes that together form a complete set of chemical processes in the cell. Since enzymes are proteins encoded on the genome, and our biochemical knowledge of metabolic reactions has a long and rich history, it is possible to reconstruct a draft metabolic network largely from genomics data alone. Tools exist that automate this process and generate a complete metabolic network (Henry et al., 2010). In general, such automatic methods can be opaque and additional physiological knowledge considerably aids in curating the resulting metabolic model (Francke et al., 2005; Notebaart et al., 2006).

**Figure 2:**
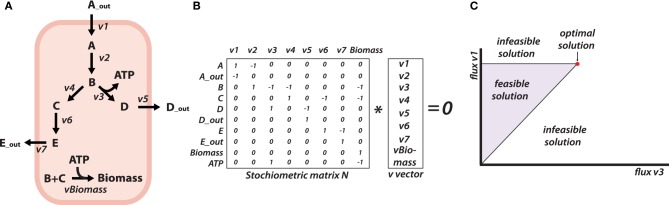
**Illustration of FBA (A)**. Visualization of a simplified metabolic network of a micro-organism. The microorganism takes up metabolite A and produces biomass, and products D and E. **(B)** The stoichiometric matrix N representing the network depicted in A, with rows corresponding to metabolites and columns to fluxes. The stoichiometric matrix multiplied with the flux vector is in steady-state always 0. **(C)** When optimization of the biomass flux is used, the (in)feasible flux distribution figure between flux v1 and v3 is calculated. The red dot corresponds with the optimal solution when the biomass flux is used as the objective function.

The topology of the metabolic network can be described by the stoichiometry of the constituent reactions. For example, consider the reaction catalyzed by the enzyme hexokinase, in a model description this would be described as: glucose + ATP ⇋ glucose-6-phosphate + ADP. This relation between reactions and reagents can be summarized for an entire metabolic network. In mathematical form, a stoichiometry matrix is created where rows represent metabolites and the columns reactions (See Figure 2B for an example).

In many applications we are interested in the behavior of a species at steady-state. At steady-state all metabolite concentrations are time-invariant, while the cells are metabolically active and consume substrate and secrete products. This implies that for each metabolite the rate of production and consumption are balanced.

In MFA, experimental data is used to deduce the true steady-state fluxes. In constraint-based modeling, FBA (see Glossary) in particular, any data that is available is combined with computational optimization techniques to predict a (in this case optimal) flux distribution through the metabolic network. Both methods have their merits and draw-backs, which we will discuss briefly.

### Metabolic flux analysis: flux from data

One of the methods used to extract more information from data is the use of MFA. MFA is a powerful tool in biotechnology and systems biology as it aims to estimate intracellular fluxes in living cells. It generally applies to systems operating at a metabolic steady-state, but is not limited to steady-state conditions. MFA tries to infer intracellular fluxes from measured extracellular fluxes using a coarse-grained metabolic model where steady-state mass balances constrain the model. In the early days of MFA, there was no standard description of how the metabolism of a cell could be defined in the stoichiometric model and therefore such descriptions could vary between models. Some defined coarse-grained metabolic blocks in which multi-reaction pathways are reduced to a single reaction, such as catabolism, respiration, product formation, anabolism, and maintenance (De Hollander, 1991). Others limited the description to central metabolism, yet included all individual reactions of central metabolism in this description (Van Gulik and Heijnen, 1995).

The more recent approaches are based on partitioning of stable-isotopic labels over intracellular metabolites (for a historical overview see Szyperski, 1998 and for a more recent review see Zamboni, 2011). ^13^C-tracer studies result in ^13^C-enrichment profiles of metabolites in the metabolism of a given strain. The ^13^C-enrichment in time indicates the amount of flux through the metabolic network. A fast enrichment indicates a high flux through the network, where a slow enrichment indicates a low flux. Exact flux values can be inferred using isotopomer-based flux analysis (see Glossary), using stoichiometric metabolic models. These models are based on biochemical data or genomic information and cover mostly the central metabolism. These approaches enable the estimation of a large fraction of the intracellular fluxes operative in central metabolism, in steady-state or dynamic states (Nöh et al., 2007; Wahl et al., 2008). The dynamic ^13^C MFA applications are still largely in development, but are perhaps most interesting to microbial ecologists because of the dynamic conditions in many environmental settings. Methods have been developed (Abate et al., 2012) and applied (van Heerden et al., 2014) to infer dynamic changes in intracellular fluxes from ^13^C tracer-experiments.

Yet, all these methods rely on data with a resolution and quantification that is still very challenging at the community level. Thus, apart from these model-aided methods to turn data into fluxes, other modeling approaches are needed: genome-scale stoichiometric metabolic models to infer fluxes largely from genomics data (Duarte et al., 2004; Teusink et al., 2009; Orth et al., 2011).

### Genome-scale stoichiometric models: flux from model exploration

Genome-scale metabolic models contain the coupled metabolic reactions encoded by the genome of an organism (Francke et al., 2005; Feist et al., 2008) (see simplified representation of a metabolic network in Figure 2A). These models tend to be much larger than the stoichiometric models used for MFA, as their purpose is to be an inventory of the metabolic potential of an organism based on its genome, not a tool *per se* for inferring fluxes from data (smaller models allow for a better estimation of fewer unknowns).

An average-sized genome-scale model consists of hundreds of reactions (Teusink et al., 2009), whereas a large model can easily contain more than thousand reactions (Herrgård et al., 2008; Thiele et al., 2013). Such large models rely on exploration and optimization for hypothesis generation and flux predictions.

Among the methods used to explore genome-scale metabolic models, FBA is by far the most widely-used method. The idea behind FBA is to find solutions that satisfy some optimal behavior of the metabolic network at steady-state; most often the maximization of biomass yield of a microorganism is used as a proxy for fitness (Orth et al., 2010). To find this optimal behavior, the metabolic network has to be constrained as much as possible (Price et al., 2004). Apart from the mass-balancing constraint that defines the steady-state condition, bounds on flux values of reactions are used as additional constraints. For instance, known irreversible reactions (derived from thermodynamic considerations) are constrained to solely positive or negative flux values. Additionally, measured substrate uptake rates and product secretion rates are also used as bounds on flux values. Other physiological characteristics of the microorganism, such as the non-growth and growth associated maintenances, the biomass composition and P/O ratio help to further constrain the model. It is also possible to obtain these physiological characteristics by exploiting the genome-scale stoichiometric metabolic models (Feist et al., 2006; Mahadevan et al., 2006). Additionally, any data that can be turned into a constraint, can be integrated into these models and bring the predicted phenotype closer to the real phenotype. Protein and mRNA data, for example, can help to improve the understanding of the fluxes through the metabolic network (Blazier and Papin, 2012; Lee et al., 2012; Reed, 2012), but also metabolomics data in combination with thermodynamic information on Gibbs energies of the reactions (Kümmel et al., 2006). The principle of FBA is shown in Figure 2C, where due to the use of a simple network, indeed a single optimal solution is obtained. FBA on metabolic networks of microorganisms generally reveals that even when microorganisms are behaving optimally, many internal flux distributions can enable this optimum. Various algorithms have been developed to calculate the flexibility of the network at its optimal state (Mahadevan and Schilling, 2003; Kelk et al., 2012). This flexibility is indicative for the possible phenotypes an organism could attain.

Using only stoichiometry and no dynamics appears a shortcoming of FBA, especially considering the dynamics in species abundances and activities in many ecosystems. However, dynamics of extracellular nutrients and biomass can be implemented in FBA. In such a case, Monod-type kinetics for uptake processes of substrates are used to constrain the uptake flux in the stoichiometric model, which through FBA will subsequently produce an optimal growth rate under that constraint. Resultant rates of uptake and growth can be integrated into a system of mass balances that constitute a population dynamic model. Microbial dynamics during batch cultivation have been described by using this so-called dynamic FBA (Mahadevan et al., 2002).

Genome-scale stoichiometric modeling of fluxes through metabolic networks have had an huge influence on biotechnology, contributing to the understanding of the physiology of industrial microorganisms and identifying targets for metabolic engineering to improve their performance. This modeling approach has been reviewed in several excellent reviews (Raman and Chandra, 2009; Gianchandani et al., 2010; Santos et al., 2011; McCloskey et al., 2013). We have listed a few relevant achievements of constraint-based modeling in the field of systems biology and biotechnology in Table 1.

**Table 1:** **Achievements of genome-scale stoichiometric modeling in systems biology**.

**Achievements**	**Reference**
First genome-scale model	Varma and Palsson, 1994
Models showed consistency with experimental data	Feist et al., 2006; Teusink et al., 2006; Oh et al., 2007
Simulations in correspondence with ^13^C-tracer studies	Sun et al., 2009
Non-optimal growth of strains	Ibarra et al., 2002; Teusink et al., 2009
Correct gene knock-out strategies for higher product yields	Bro et al., 2006; Pharkya and Maranas, 2006; Park et al., 2007; Lee et al., 2007

## Modeling in bacterial communities

How can we use the modeling methods for monocultures for microbial communities? To be unambiguous about the inference problem at the community scale, let's take a more formal perspective on what we mean by community structure and the community state (see also Glossary). The *community structure* implies the ordering of microorganisms through interactions, ending up in a connected network. It provides the topology of the network, much like the stoichiometry does for a metabolic network. In theory, inference methods can be used to identify such community structure, by measuring the *community state* over time (Gonzalez et al., 2012).

At any given moment in time, this community state is characterized by: (i) the abundances of all microorganisms in the community; (ii) the intracellular and extracellular concentrations of their metabolites, mRNAs, enzymes, and other cellular constituents; and (iii) the process rates (fluxes), i.e., the growth rates of all microorganisms, and the rates of all the intracellular and extracellular biotic metabolic reactions and abiotic processes. Thus, the community state describes the quantitative values of all the concentrations and process rates occurring in the community. For single species cultures, such a data set could be acquired but already constitutes a major task. There are huge experimental complications for microbial communities, and this makes the inference of the community structure also challenging. In theory, if we were to have a kinetic representation of all the processes, the characterization of the community state would suffice to determine the rates of change of all the concentrations in the community. Clearly, we do not know most of the kinetic parameters that would be required. This inference problem is still an enormous challenge in modeling monocultures (Link et al., 2014). As a result, we have to constrain the description of the community system greatly, simplify smartly, and set realistic goals.

In ecosystems, the community-level fluxes that are measured correspond to the sum of cell-specific reaction activities multiplied by their respective abundances. Flux analysis of communities therefore requires the weighing of fluxes by biomass abundances of the community members (Khandelwal et al., 2013). This makes flux analysis for a community more complicated than flux analysis for single microorganisms. The inference method that we envision will, therefore, involve the measurements of community-level fluxes, abundances of species and knowledge of the metabolism of these organisms. This requires the consideration of all the metabolic fluxes in the community, which in complex ecosystems can amount to millions of reactions—many of which can be the same reaction but carried out in different species. The constraints imposed on the network do decrease the amount of solutions in community modeling considerably. Even so, sophisticated computer models will be required to infer knowledge from microbial community data.

This situation is reminiscent of early-days MFA, where only partial genome information and data on extracellular fluxes were available. For communities the challenges are clearly much larger. FBA-type simulations on the metabolic network models of species in a microbial ecosystem will be needed to fill in inevitable gaps in our data sets. These models can integrate the available meta-omics data of the ecosystem with knowledge about reaction stoichiometries and thermodynamics. Such type of metabolic models can provide detailed insights into the metabolic capacities of single microorganisms in multispecies settings and can be extended to deal with communities (Zengler and Palsson, 2012). They provide a straightforward tool for biologists to integrate data and make realistic predictions, given constraints that derive from basic principles and experimental data. They have been successfully used to understand processes in synthetic ecosystems (Stolyar et al., 2007), but could also explain phenotypic behavior in real ecosystems (Zhuang et al., 2010).

### Genome-scale modeling approaches in bacterial communities

We therefore believe that the application of stoichiometric models of the coupled metabolisms of microorganisms in communities holds great promise, for several reasons:

1. CSSMs (see Glossary) are very suitable for data integration, as their mathematical description directly maps onto genomic, metabolomic, proteomic, and flux data of the metabolism of individual microorganisms in the community. The mapping can be done on a visualized metabolic map corresponding to the microbial ecosystem according to methods described in Maarleveld et al. (2014).
2. CSSMs allow for calculation of the community state and structure with numerical algorithms.
3. Experimental data can be used as constraints in the associated modeling formalism, to improve predictions when more data has become available (Röling and Van Bodegom, 2014).
4. The systemic consequences at community scale of molecular or physiological perturbations or species augmentation can be explored with CSSMs.
5. CSSMs can be used for experimental or medium design to improve community performance.

Different approaches to extend single-species models to microbial-community metabolic network reconstructions have been proposed, each trying to answer different research questions. The existing applications of FBA to CSSMs can be classified into three different groups: (i) the supra-organism approach (Rodríguez et al., 2006), (ii) the steady-state compartmentalized approach (Stolyar et al., 2007; Khandelwal et al., 2013), and (iii) the dynamic compartmentalized approach, based on dynamic FBA (dFBA) (Salimi et al., 2010; Zhuang et al., 2010; Hanly and Henson, 2011). These methods vary in the complexity of the CSSM description and how they choose to handle individual species.

The supra-organism approach has first been applied by Rodríguez et al. (2006). It combines all metabolic reactions of the various species in the microbial ecosystem to create a single meta-metabolic network to study the metabolic capacities in terms of product and substrate variation of the community. This approach simplifies the complexities of interactions and regulations amongst cohabiting species and makes it easy to predict environmental conditions that can be imposed to optimize a community level objective toward an outcome of interest to biotechnology. By combining all reactions into one network, it ignores the impact of species abundances and the interactions between community members.

The steady-state compartmentalized approach considers the various species as separate compartments where one shared compartment is introduced for the exchange of metabolites between the species. This approach elucidates interactions between species, contributing to an improved insight on host-microbe/pathogen interactions or mutualistic interactions. With this approach, for instance, it is suggested that instead of formate, H_2_ is exchanged in a co-culture of *Desulfovibrio vulgaris* and *Methanococcus maripaludis* (Stolyar et al., 2007). Initial compartmentalization approaches neglected biomass concentrations of each species individually, resulting in biased quantitative flux distributions. Implementation of biomass concentrations in the compartmentalization approach has recently been successfully applied and is of particular interest when accurate quantitative transfer rates are required (Khandelwal et al., 2013).

The dynamic compartmentalized approach implements dFBA by using the kinetics of substrate uptake and metabolite exchange between species (Salimi et al., 2010; Zhuang et al., 2010; Hanly and Henson, 2011). Implementation of dynamic behavior is often required to understand ecosystem structure and functionality. Here, biomass concentration of each species is taken into account and can change with time. Therefore, it is possible to simulate competition, predation or other interactions altering the community state. The requirement of quantitative kinetic information is one of the major disadvantages of the dFBA method. Obtaining this for every species in a community will be laborious, if at all possible. However, the kinetic parameters can be inferred by fitting the model with experimental data.

A limitation of all these methods is that they are currently tailored for simulation and not for inference of metabolic activity and abundances of microorganisms from community-level fluxes. Even though such models can actually be used for this purpose, this is rarely done, because of experimental challenges in obtaining the data. In particular resolving fluxes at the level of the single species remains challenging. However, recently species level isotopomer-based flux data were obtained from synthetic consortia (Shaikh et al., 2008; Rühl et al., 2011). When experimental procedures for isotopomer-based analysis of fluxes through microbial communities are developed further, the CSSMs as we describe here, also become relevant to experimentalists. Moreover, recent work (Niebel, Heinemann, personal communication) provides hope that detailed thermodynamic constraints, which are physical constraints that should apply to all organisms, may sufficiently constrain metabolic networks to infer realistic intracellular flux distributions without the need for isotopic labeling data. Thermodynamic constraints can also contribute to the search for novel microorganisms that can carry out biochemical conversions in nature that appear thermodynamically possible on paper (Raghoebarsing et al., 2006). In such work, initially a coarse-grained perspective is taken to understand overall metabolic potentials in ecosystems, and this approach is supplementary to the genome-scale approaches discussed so far.

### Inferring the community structure and state using coarse-grained models

**Figure 3:**
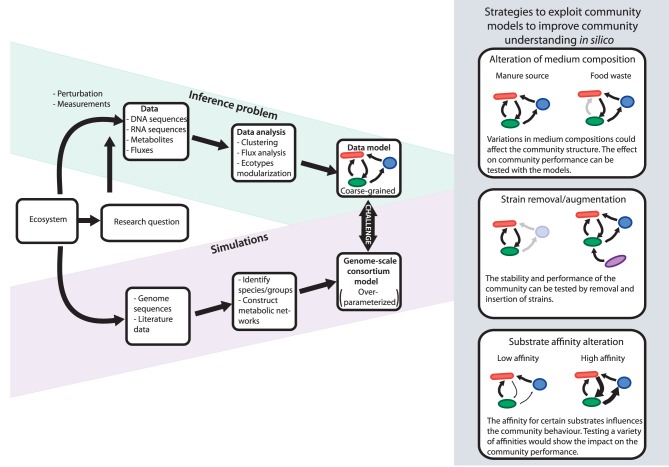
**Representation of the work flow to get more information from experimental data**. On one hand the inference approach with as result a coarse-grained data-model which fits the measured data and on the other hand the genome-scale models which are highly underdetermined, but can be used as data repositories. The challenge is to create models which fit the research question. This can be done via the represented steps.

Even though CSSMs can be very useful, for many applications they will be too detailed and too unparameterized to be of immediate value. The details will result in many degrees of freedom, for example in many potential substrates and products. A more pragmatic approach is therefore often needed. Simplifying and coarse-graining models is also regularly required for models of monocultures. This happens when the data is still insufficient to parameterize a model, or when the model becomes so detailed that it hampers understanding. Even well-curated models might be too detailed and complicated to understand the simulated results, because there are too many variables and independencies. In such cases there may be predictive power but no understanding. Obviously, it depends on the question what is preferred: ideally, the type of research question determines the required level of detail and initially limits the complexity of the model, at the same time allowing for progressively and gradually increasing the model in size when more experimental data becomes available. The work flow we envision is visualized in Figure 3.

The coarse-grained models can be created at different levels of granularity. However, in order to model microbial communities, we envisage that a more detailed (but unparameterized) description, at a lower level of granularity, is always possible. Ultimately, there is the genome of the individual consortium species that allows for a reconstruction of the metabolic potential, which is a solid ground on which to build more detailed reconstructions. When the genome of the consortium species is not present, data from metagenomics is suitable for more detailed metabolic reconstructions. However, the level of detail of the metabolic reconstructions will be largely dependent on the sequencing depth of the metagenome.

## Application to a case study

We will make use of an elegant study performed on a nitrate respiring community (Kraft et al., 2014) as an example to illustrate and explain how the proposed work flow could be applied in practice.

**Figure 4:**
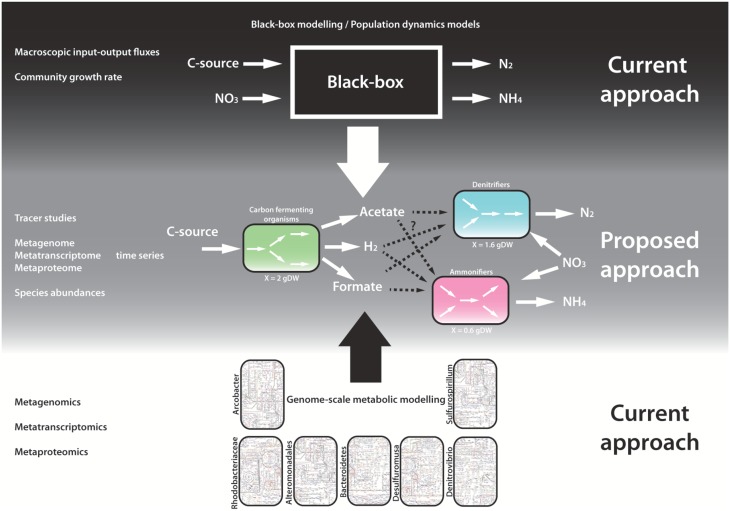
**Illustration of the models used to get a mechanistic understanding of the nitrate respiring community studied by Kraft et al. (2014)**. At the top the coarse-grained models, whereas at the bottom the highly detailed genome-scale models are shown. In the middle is the type of model created using the synergistic approach of the coarse-grained models and the genome-scale models which will contribute to mechanistic understanding of the nitrate respiring community and it's functioning.

Kraft et al. (2014) incubated a nitrate respiring community, originating from sediment in the German Wadden Sea, in reactors with continuous substrate supply, and altered environmental factors to investigate if denitrification or ammonification evolved as the major nitrate respiring process. The researchers measured community level fluxes, performed metagenomics, metatranscriptomics, and metaproteomics, and used fluorescence *in situ* hybridization to unravel the effect of specific environmental conditions on community behavior. A schematic overview of the metabolic interactions was created, based on the meta-omics data, with fermenting microorganisms generating fermentation products that were consumed by the microorganisms respiring nitrate to either ammonium (ammonification) or nitrogen gas (denitrification) (see Figure 4). In total 7 dominant populations related to ammonification and denitrification were identified. Denitrification became dominant at short generation times and with nitrite supply, while nitrate supply and long generation times with nitrite as supply favored ammonification. The same enzymes are responsible for the first steps in both processes, which branch after nitrite reductase. Therefore, they hypothesized that differences in apparent affinity of this enzyme for nitrite determines the dominating respiration process, with the nitrite reductase of denitrifying microorganisms having higher affinities.

The study by Kraft et al. (2014) covers the “Data” and the “Data analysis” part of the “Inference problem” in Figure 3, but several hypotheses and open questions could not be answered by the data. This study therefore provides an interesting test case for integration of the experimental data into a mathematical model to get mechanistic understanding of community functioning and eventually could lead to control and steer its performance. The third and last step in our proposed work flow in Figure 3 of the “Inference problem” would be the construction of a coarse-grained data model describing a limited set of pathways and connect them to each other to create a community (Figure 4). Once the simulations with the coarse-grained models match the experimental data, they can be applied to answer questions about the community. The type of research question will influence which parts of models should be described in high detail and which not.

For instance, to address the hypothesis that nitrite affinity determines the major respiration process, we start by creating simple models of the three functional groups (ammonifiers, denitrifiers, and carbon fermenting organisms). The model of the carbon fermenting organism will be the simplest, that is: uptake reactions of glucose and amino-acids and secretion reactions of acetate, formate and hydrogen, because testing the given hypothesis only requires the secretion of fermentation products that can act as substrate for the other functional groups. The ammonifiers model should contain the metabolic pathway of nitrate to ammonium conversion, transport reactions, carbon metabolism and other energy generating pathways. The denitrifiers require the same level of detail in their model as the ammonifiers. Now, these simple coarse-grained models allow to first investigate whether the experimentally observed differences might be explained without requiring the inclusion of the affinities for nitrite. If not, a kinetic description of the nitrite reductase in both functional groups can be included. Subsequently, it is possible to determine how big the difference in nitrite affinity should be to explain the experimental results.

Kraft et al. (2014) also observed large differences between the potential rates of nitrite reduction between the ammonification and denitrification process, in particular when multiple electron donors were fed to the consortium. They hypothesized that this could be caused by a bottleneck in electron supply to the nitrite reductases of ammonification. Those proteins require six electrons per nitrite, where only one electron is required for denitrification. Testing this hypothesis requires an additional level of detail compared to the models presented in the first example: the metabolic pathways of the different electron donors should now be implemented. A medium with multiple electron donors can be simulated and compared with a medium with only one electron donor. The model will show whether the ammonifiers become relatively less active when multiple electron donors are provided to the community compared to a situation in which one carbon source is provided.

Finally, Kraft et al. (2014) observed functional redundancy within both denitrifiers and ammonifiers, and the applied generation time determined which population became dominant within these groups. To better understand the underlying mechanisms, a more detailed model would be required compared to the previous examples. Firstly, each functional group needs to be split in several populations, each capable of either ammonification or denitrification. Secondly, for each population a more detailed metabolic model needs to be constructed, to capture differences in metabolism within the functional groups. These models are informed by the binned metagenomic data of Kraft et al. (2014). The resulting models could subsequently be tested. To establish what causes the difference in dominance in relation to generation time, kinetic information to describe competition should likely be included.

The aforementioned examples show how the question determines the level of detail of the “Inference models.” These coarse-grained models of the players in the ecosystem should be based on knowledge of the (genome-derived) metabolic networks of the species. Therefore, parallel to this “Inference model,” genome-scale stoichiometric metabolic models are maintained to act as data repositories (shown in Simulation in Figure 3). For the key species in the community, genome-scale metabolic reconstructions could be created using genomic, literature, and experimental data. All models can be combined in one metagenome-scale compartmentalized consortium model and allow for the exploitation and exploration of the microbial community.

Finally, a synergistic approach emerges where the interplay between the simple coarse-grained models and the genome-scale metabolic models are determined by the type of question being asked about the community. An important aspect is that the output of models with different levels of description should be consistent with each other and agree with available data. This process of iterative model making should inform the experimentalists about knowledge gaps and suggests new experiments.

As demonstrated by Kraft et al. culturing communities under well-controlled conditions, for instance in chemostats, enables quantification of community-level fluxes, biomass abundances and gene expression levels of specific populations, and allows for applying well-tractable perturbations. This type of experimental approach will be very helpful to develop and optimize methods for species-specific flux descriptions on basis of CSSMs, to understand the community function and eventually control the microbial ecosystem.

## Concluding remarks

In this paper, we described current methods to model the metabolism of single species and how we think such system approaches could be used at the microbial community level. These methods go from genomic information to understanding and directing microbial community metabolism. We argued that metagenomics data are great, but they need to be augmented with flux measurements and model-based inference methods to identify the community structure and state. We arrive at a proposed approach where models are combined with quantitative data. Such models can vary in their level of coarse-graining depending on the research questions, data and experimental options at hand. Genome-scale stoichiometric models will be the knowledge base with which to integrate genomic, physiological and physicochemical data, and can be developed parallel to coarse-grained models to understand the community structure, state and function. We feel that what is missing -but is within reach- is a description of the functioning of a microbial community in terms of the fluxes through its members. We therefore advocate that flux analysis specialists from the fields of biotechnology, microbial physiology, and systems biology team up with microbial ecologists, as they increasingly do.

### Conflict of interest statement

The authors declare that the research was conducted in the absence of any commercial or financial relationships that could be construed as a potential conflict of interest.

## Glossary

*Community-scale stoichiometric model (CSSM):*: Stoichiometric model that describes the microbial community.
*Community state:*: The full set of concentrations, abundances of species and process rates within a community.
*Community structure:*: Topology of the network of species interactions, as selected by the environment.
*Flux Balance Analysis (FBA):*: Method used to calculate flux distributions through the cell. Measured flux data are not required, so FBA can be used purely computational. FBA is performed on genome-scale stoichiometric metabolic networks.
*Isotopomer:*: Molecules which have the same constitution and the sameconfiguration but differ in isotope substitution.
*Isotopomer-based flux analysis:*: Advanced method for Metabolic Flux Analysis, using isotopomer data to infer intracellular fluxes from isotopomer enriched metabolites.
*Metabolic Flux Analysis (MFA):*: Method to infer fluxes from measured flux data. The stoichiometric models are coarse-grained and are used by the experimentalists.
